# Early iron-deficiency-induced transcriptional changes in *Arabidopsis *roots as revealed by microarray analyses

**DOI:** 10.1186/1471-2164-10-147

**Published:** 2009-04-06

**Authors:** Thomas J Buckhout, Thomas JW Yang, Wolfgang Schmidt

**Affiliations:** 1 Institute of Biology, Humboldt University Berlin, Invalidenstraße 42, 10115 Berlin, Germany; 2 Institute of Plant and Microbial Biology, Academia Sinica, 115 Taipei, Taiwan

## Abstract

**Background:**

Iron (Fe) is an essential nutrient in plants and animals, and Fe deficiency results in decreased vitality and performance. Due to limited bio-availability of Fe, plants have evolved sophisticated adaptive alterations in development, biochemistry and metabolism that are mainly regulated at the transcriptional level. We have investigated the early transcriptional response to Fe deficiency in roots of the model plant Arabidopsis, using a hydroponic system that permitted removal of Fe from the nutrient solution within seconds and transferring large numbers of plants with little or no mechanical damage to the root systems. We feel that this experimental approach offers significant advantages over previous and recent DNA microarray investigations of the Fe-deficiency response by increasing the resolution of the temporal response and by decreasing non-Fe deficiency-induced transcriptional changes, which are common in microarray analyses.

**Results:**

The expression of sixty genes were changed after 6 h of Fe deficiency and 65% of these were found to overlap with a group of seventy-nine genes that were altered after 24 h. A disproportionally high number of transcripts encoding ion transport proteins were found, which function to increase the Fe concentration and decrease the zinc (Zn) concentration in the cytosol. Analysis of global changes in gene expression revealed that changes in Fe availability were associated with the differential expression of genes that encode transporters with presumed function in uptake and distribution of transition metals other than Fe. It appeared that under conditions of Fe deficiency, the capacity for Zn uptake increased, most probably the result of low specificity of the Fe transporter IRT1 that was induced upon Fe deficiency. The transcriptional regulation of several Zn transports under Fe deficiency led presumably to the homeostatic regulation of the cytosolic concentration of Zn and of other transition metal ions such as Mn to avoid toxicity.

**Conclusion:**

The genomic information obtained from this study gives insights into the rapid transcriptional responses to Fe shortage in plants, and is important for understanding how changes in nutrient availability are translated into responses that help to avoid imbalances in ion distribution. We further identified rapidly induced or repressed genes with potential roles in perception and signaling during Fe deficiency which may aid in the elucidation of these processes.

## Background

Iron is essential for numerous biological oxidation-reduction reactions in plants and plays a central catalytic role in all primary metabolic processes, including chlorophyll biosynthesis, photosynthetic and respiratory electron transport, nitrogen assimilation, and a large number of other anabolic and catabolic reactions [1]. In an oxidizing environment, the concentration of soluble Fe^3+ ^is low and the Fe that is present is available to the cell typically in the form of ferric chelates. The mechanism for Fe uptake in *Arabidopsis *and all other dicotyledonous and non-grass monocotyledonous plants involves the reduction of Fe^3+^, release of Fe^2+ ^from a chelate, and uptake of the liberated Fe^2+ ^by the cell (strategy I; [2,3]). In *Arabidopsis*, the plasma membrane-bound Fe^3+^-chelate reductase FRO2 catalyzes the reduction of Fe^3+ ^at the cell surface [4]. Homologues of *FRO2 *have been found in other strategy I plants such as pea [5], tomato [6], and cucumber [7]. The Fe^2+ ^is then taken up by an Fe-deficiency-regulated transporter of the ZIP family, IRT1 [8,9]. In addition to transcriptional regulation, IRT1 and FRO2 are subject to posttranslational regulation, indicating the importance of an exact and rapid response by adjusting the amount of protein appropriate to the Fe demand in order to avoid the accumulation of surplus amounts of Fe [10,11]. The coordinated functions of IRT1 and FRO2 are principally responsible for Fe uptake in *Arabidopsis *roots [4,12,13].

Research in the regulation of the Fe deficiency response in plants has generated intense interest. The first insights into the nature of this regulation were based on the early discovery of the *fer *mutant in tomato [14]. The *fer *mutant (t3238*fer*) was unable to initiate the typical responses to Fe deficiency, including enhanced extrusion of protons, proliferation of root hairs, and Fe^3+^-chelate reductase activity. Furthermore, relatively low Fe concentrations (e.g. 0.1 μM) in the growth media were lethal [15]. The *FER *gene encodes a bHLH transcription factor that is expressed in roots and root tips but not in the leaves or upper hypocotyls [15]. The expression of *FER *was not greatly altered in tomato plants grown with 0.1 or 10 μM Fe. Thus in tomato, *FER *is expressed in a root-specific and largely Fe-independent manner.

In *Arabidopsis, FIT *(AtbHLH029) was discovered as a *FER *homolog responsible for regulating the Fe deficiency response [16-19]. The genes *FRO2 *and *IRT1 *were found to be regulated by FIT, which also controls the expression of a total of 72 genes with known or putative functions in Fe homeostasis [16]. The transcriptional factor FIT was itself regulated by Fe deficiency; however, constitutive expression of *FIT *under the control of a 35S promoter did not alter the expression patterns of *IRT1 *and *FRO2*, nor was the Fe content in the *FIT *over-expressing plants altered compared to controls [16]. It was concluded that FIT is necessary but not sufficient for regulation of the Fe deficiency response in *Arabidopsis*. Recently, it was demonstrated that FIT interacts with two bHLH transcription factors, AtbHLH038 and AtbHLH039 [20]. The over-expression of *FIT *with either AtbHLH038 or AtbHLH039 resulted in the constitutive expression of both *IRT1 *and *FRO2 *and the increased accumulation of Fe compared to controls. The function of AtbHLH038 and AtbHLH039 was shown by T-DNA insertion mutations to be redundant [21]. These results have led to the conclusion that an as yet unidentified *cis*-element is responsible for the Fe-deficiency-associated regulation of *IRT1 *and *FRO2 *among other genes.

As in *Arabidopsis*, the mechanism of perception of Fe deficiency in grasses is still unknown. The c*is*-acting elements IDE1 and IDE2, which are presumably responsible for Fe-deficiency-inducible expression, have been identified in the promoter region of the barley IDS2 gene [22]. A constitutively expressed transcription factor, IDEF1 belonging to the ABI3/VP1 family, has been shown to specifically bind to IDE1, and over-expression of *IDEF1 *led to the induction of the bHLH transcription factor OsIRO2 [23]. *OsIRO2 *expression was also induced by Fe deficiency, and OsIRO2 itself has been shown to bind to the *cis*-element 5'-CACGTGG-3' [24]. This *cis*-element was found in the promoters of a number of genes involved in Fe uptake in grasses. In particular, OsIRO2 was shown to be required for the transcriptional regulation of the genes involved in phytosiderophore synthesis and two transcription factor genes, OsNAC4 and a gene encoding an AP2 domain-binding protein. These later two transcription factors appeared to be regulated directly by OsIRO2 [25].

Recently, a transcription factor belonging to the NAC family, IDEF2, was found to bind specifically to the IDE2 element [26]. NAC transcription factors are a plant-specific family, which have been implicated in developmental responses to biotic and abiotic stress [27]. IDEF2 transcripts were constitutively expressed in shoots and roots and were not greatly altered under Fe deficiency. Reduction of IDEF2 transcript abundance by RNAi under conditions of Fe deficiency resulted in a considerable reduction in transcript abundance of the Fe-phytosiderophore transporter OsYSL2. At present, it appears that in grasses, the Fe-deficiency signal is mediated by at least two constitutively expressed transcription factors that under conditions of Fe deficiency, initiate a transcriptional cascade of events that lead to the adaptive responses to Fe deficiency.

There are similarities in the response pathway to Fe deficiency in *Arabidopsis *and rice (e.g. IDE1-like sequences and involvement of bHLH transcription factors), and based on our current knowledge, it is reasonable to expect a higher complexity in the response chain in *Arabidopsis*. The elucidation of regulatory components is rendered more difficult by the lack of knowledge on the temporal pattern of their expression. We present here a detailed time-course analysis of the short-term transcriptional changes induced by Fe deficiency by using DNA microarrays. We demonstrate that the majority of transcriptional changes were established within the first six hours after subjecting the plants to Fe deficiency. The transcriptional changes include most of the previously reported transcriptional changes in response to Fe deficiency but also novel transcripts that are likely to be important in transduction of the Fe deficiency response in *Arabidopsis*.

## Results

The characterization of rapid responses to Fe deficiency at the transcriptome level requires a growth system that permits rapid removal of Fe from the media. Recently, various groups have published DNA microarray experiments using plants grown on agarose plates. Initiation of Fe deficiency was accomplished by transferring to Fe-deficient media, often in the presence of a Fe^2+ ^chelator. Although widely used, the inherent drawbacks of the agar plate system include, inducing physical injuries to the plants during transferring, the roots carry-over of residual amounts of Fe-containing agarose, thus making the time-point of Fe deficiency difficult to determine, and lastly, the use of chelators that may affect the partitioning of metals across the plasma membrane independent of Fe deficiency. In the present study, we have used a well-characterized hydroponic system to analyze the Fe-deficiency response [28]. The system allowed the bulk transfer of a large number of plants with intact roots into Fe-sufficient or Fe-deficient media within seconds and with minimal mechanical damage. Our approach allows for a time-dependent monitoring of Fe deficiency-induced transcriptional changes with a clear-cut onset of the exposure to Fe-free media. We have used this system to generate materials used in the present study.

### Time-course of Fe deficiency-induced alterations in transcript abundance

**Figure 1:**
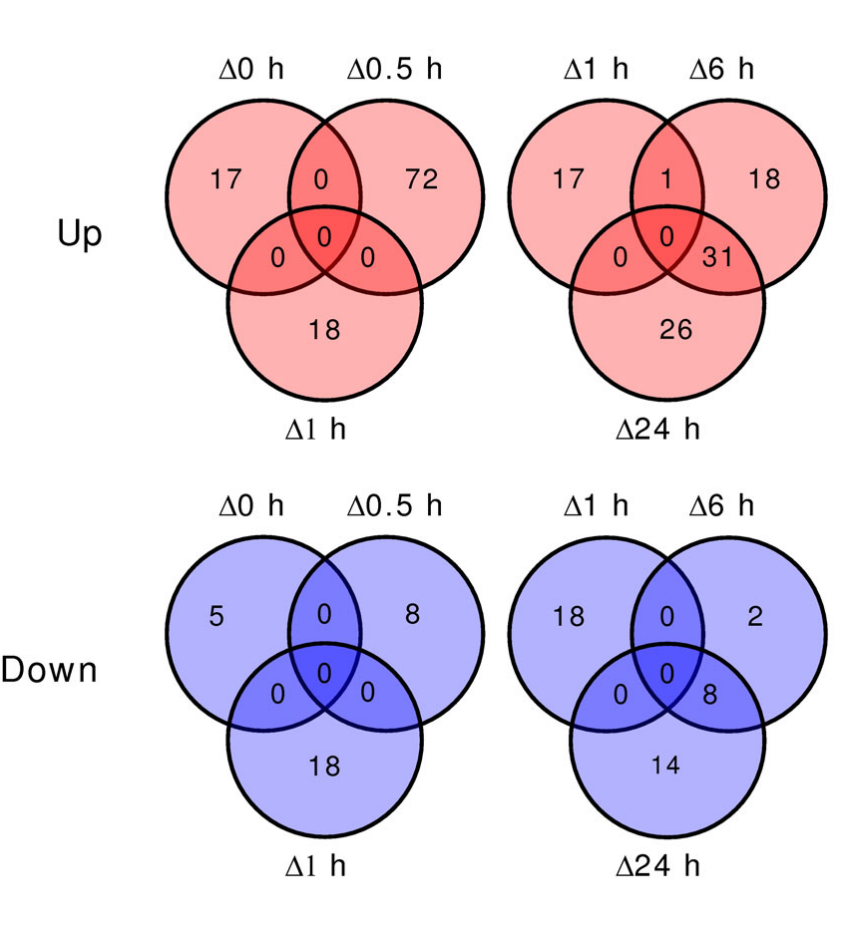
**Venn diagrams summarizing time- and Fe-dependent changes of transcript abundance in *Arabidopsis *roots**. The intersections of the circles for the individual time points represent the number of genes whose transcript abundance with respect to the control treatments was increased (red, "up") or decreased (blue, "down"). For time-dependent and Fe-dependent changes, gene transcripts were tallied whose *p*-score was < 0.05.

To characterize the early response of transcript abundance to the removal of Fe from the growth media, differential gene expression was determined by DNA microarray analysis following 0, 0.5, 1, 6 and 24 h of Fe-deficient growth. For an initial characterization of the response to Fe deficiency, the genes were extracted from the data base that had a *p*-value of < 0.05 and a fold change of > 50%. These results are summarized in Venn diagrams for up- and down-regulated transcripts at very early (0 to 1 h) and early (1 to 24 h) time-points following removal of Fe (Figure 1). With only one exception, changes in transcript abundance at 0, 0.5 and 1 h were limited to a single time point, and the differentially expressed genes that were detected immediately after transfer or after 0.5 or 1 h of Fe-deficient growth were not persistent over time. An examination of the over- or under-represented gene ontogeny categories (GO) showed no categories that were indicative of a response to Fe deficiency (Table 1). For these reasons, the genes showing differential expression at the 0, 0.5 and 1 h time points were considered to be Fe-unspecific responses.

**Table 1:** A summary of over-represented GO categories of genes responding to Fe deficiency^a^

Treatment (h, -Fe)	DEG	Gene Category	*p *value, corrected
0	22	none	
			
0.5	80	response to light stimulus	1.7e-02
		carbon utilization	3.6e-02
			
1	36	sugar binding	3.1e-02
			
6	60	transporter activity	7.1e-02
		iron ion binding	2.4e-04
		response to metal ion	1.3e-03
		cation transport	2.6e-02
			
24	79	iron ion binding	3.83e-03
		ion transporter activity	6.83e-03
		C-C lyase activity	3.43e-02

^a ^Transcript intensity was determined and differentially expressed genes (DEG) with an uncorrected *p *< 0.05 and a change in intensity between Fe-sufficient and Fe-deficient treatments of 50% were selected for each of the five time points analyzed. Biological processes and molecular functions were determined as being over-expressed when a corrected *p *< 0.05 was determined. The correction for false discovery rates was determined with Bingo software using the method of Benjamini and Hochberg [51].

Based on the total number of genes analyzed, approximately 0.3% of the transcripts were changed in abundance after 24 h of Fe-deficient growth. Furthermore, approximately 50% of the transcripts that showed a significant change in abundance after 24 h were already differentially expressed by 6 h (Figure 1). The GO categories that contained more than two members and that were over-represented at 6 and 24 h Fe deficiency were associated with cation transport processes and Fe homeostasis (Table 1). The 40 gene transcripts that were unique to the 24 h time-point showed no over-representation in ontogeny categories; whereas the transcripts unique to 6 h showed over-represented gene ontogeny categories for Co/Zn detoxification, nicotianamine metabolism and Cu transport (data not shown). It was, however, clear that the early response to Fe deficiency was initiated between 1 and 6 h of growth under Fe-deficient conditions.

### Clustering of Fe-deficiency changes in gene expression

**Figure 2:**
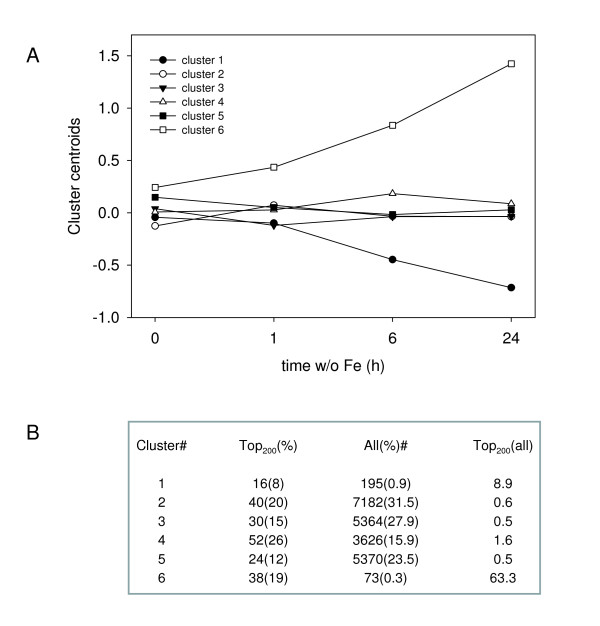
**The averaged response of clusters to Fe deficiency**. The entire array dataset was subjected to a k-means cluster analysis, and the centroids of these clusters were plotted with respect to time (A). The misrepresentation of significantly expressed gene transcripts is reported below the figure (B). For this analysis, the total frequency of genes found in a cluster was compared to the transcript frequency in the 200 statistically most significant signals.

To further characterize the response to Fe deficiency in a time-course manner, all data for transcript abundance at 0, 1, 6 and 24 h Fe deficiency were subjected to a cluster analysis using k-means methods with the algorithm as described previously [29]. The cluster stability was determined using the "benhur" function found in the clusterStab package of the Bioconductor software . Apart from a two-cluster model that was not useful in analyzing the response to Fe deficiency, a six-cluster model generated the smallest number of clusters that gave the most stable results following multiple resorting. Therefore, the six-cluster model was used for further analysis. Clusters one and six showed transcripts with decreasing and increasing abundance during Fe-deficient growth, respectively (Figure 2). The significance of these trends was confirmed by a comparison of the transcript frequency in the top 200 statistically most altered transcripts with the frequency of transcripts in each cluster. The transcripts in cluster one and six were approximately 9- and greater than 60-fold overrepresented, respectively. Cluster four was slightly overrepresented in the top 200 transcripts, while the remaining clusters were slightly underrepresented. Clusters one and six clearly contained the majority of transcripts that were responding to Fe deficiency.

**Figure 3:**
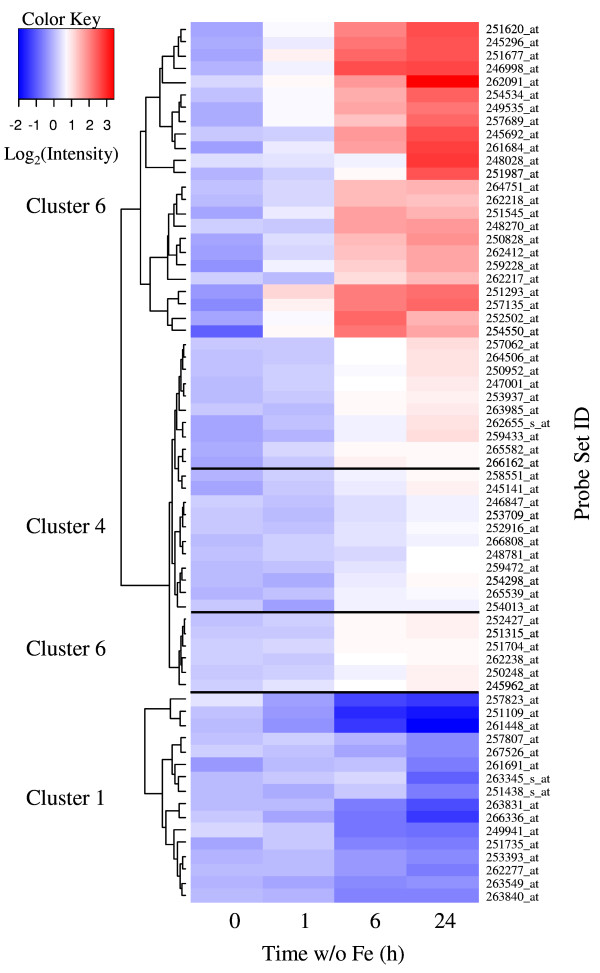
**Heatmap analysis of changes in transcript abundance in Arabidopsis roots grown under Fe deficiency**. The fine structure of clusters one and six were investigated. Changes in response to Fe deficiency with a p-value of < 0.05 were selected for analysis. In a detailed analysis, several transcripts that were grouped into cluster 4 by an analysis of all transcripts showed an overlap with cluster six. The overlapping members of cluster 4 are also shown in the figure.

**Figure 4:**
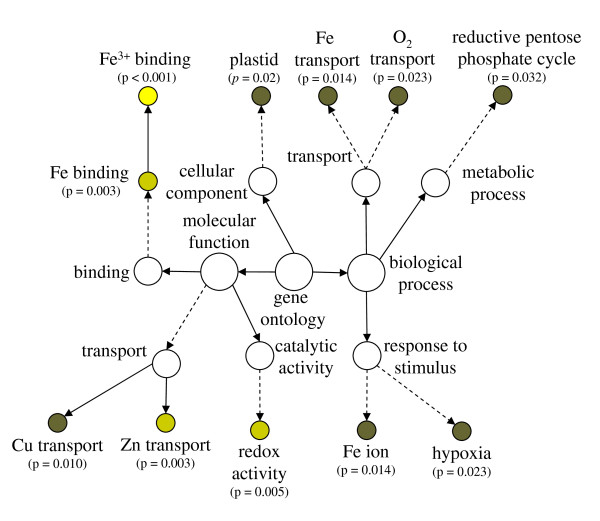
**Over representation of gene ontogeny categories (GO) in genes showing decreased abundance under Fe deficiency**. The genes, taken from Table 2, were analyzed using BinGO [50] and corrected for false discovery rate using the method of Benjamini and Hochberg [51]. Dotted lines indicate intermediate categories that are not shown.

The fine structure of clusters one and six was investigated using heatmap diagrams and hierarchical clustering methods using Euclidian distances (Figure 3). Only transcripts with a *p *< 0.05 were investigated. The transcript abundance in cluster one decreased during Fe-deficient growth. The decrease was weakly apparent after 1 h Fe-deficient growth and clearly evident at 6 and 24 h. The kinetic behavior of individual transcripts was similar, with a small group of three transcripts showing a more pronounced response (Figure 3, cluster one). Prominent among the down-regulated transcripts in this group were the three ferritin genes, bacterial-type hemoglobin (GLB3) and the ZIP3 transporter. These genes have functions in Fe storage, binding and transport (Table 2). The biological processes of Fe transport and stimulus and the molecular functions of Fe binding and Cu and Zn transport were significantly over-represented in the genes showing a decrease in abundance (Figure 4). Interestingly, an over-representation in transcripts that encode proteins presumably localized in plastids was observed. Not surprisingly however, these transcripts included the ferritin genes but also components of the reductive pentose cycle.

**Table 2:** List and description of genes transcripts down-regulated by Fe deficiency^a^

Probe ID	ATG	Description	*p*-value
257823_at	At3g25190	Integral membrane protein; putative nodulin	0.047
251109_at	At5g01600	Ferritin 1 (ATFER1)	0.000
261448_at	At1g21140	Similar to a nodulin-like protein	0.034
257807_at	At3g26650	Glyceraldehyde 3-phosphate dehydrogenase A subunit (GapA), chloroplast	0.012
267526_at	At2g30570	Photosystem II reaction center 6.1KD protein, (PsbW) protein-related	0.041
261691_at	At1g50060	Pathogenesis-related protein containing a SCP-like extracellular domain	0.038
263345_s_at	At2g05070	Putative chlorophyll a/b binding protein; LHCII type II	0.038
251438_s_at	At3g59930	Defensin-like (DEFL) protein	0.001
263831_at	At2g40300	Ferritin 4 (ATFER4)	0.001
266336_at	At2g32270	A member of Zrt- and Irt-related protein (ZIP) family(ZIP3)	0.002
249941_at	At5g22270	Expressed protein with unknown function	0.023
251735_at	At3g56090	Ferritin 3 (ATFER3)	0.007
253393_at	At4g32690	Expressed protein, 2-on-2 hemoglobin (GLB3)	0.034
262277_at	At1g68650	Expressed transmembrane protein of unknown function	0.002
263549_at	At2g21650	MEE3 (maternal effect embryo arrest 3); myb-like transcription factor	0.001
263840_at	At2g36885	Expressed protein with unknown function	0.001

^a ^Listed are the genes in cluster 1 that showed a significantly (p < 0.05) decreased transcript abundance in response to Fe deficiency. The appearance in the list (top to bottom) corresponds to the order shown in Figure 3. Gene annotation is based on the 8.0 release of the *Arabidopsis *genome.

The genes found in cluster six, which contained transcripts that responded with increasing abundance following removal of Fe, could be divided into two subgroups by hierarchical clustering of approximately equal size. The first of these responded to Fe deficiency rapidly and with high increases in abundance already after 6 h of Fe deficiency (Figure 3). A tendency for increased abundance was apparent after 1 h; although, this increase was not significant in all cases. Both of these groups contained a number of transcripts that were known to respond to Fe deficiency, including *IRT1*, *IRT2*, *FRO3 *and some bHLH transcription factors (Table 3). Genes encoding transport proteins and transcription factors were prominently represented in this group.

**Table 3:** List and description of genes transcripts strongly up-regulated by Fe deficiency^a^

Probe ID	ATG	Description	*p*-value
251620_at	At3g58060	Cation efflux family protein (MTPc3)	0.001
245296_at	At4g16370	Oligopeptide transporter (OPT3)	0.000
251677_at	At3g56980	Basic helix-loop-helix transcription factor (BHLH039)	0.006
246998_at	At5g67370	Expressed protein with unknown function	0.005
262091_at	At1g56160	R2R3 transcription factor (MYB72)	0.001
254534_at	At4g19680	Fe^2+ ^transport protein (IRT2)	0.008
249535_at	At5g38820	Amino acid transporter family protein	0.026
257689_at	At3g12820	Myb family transcription factor (MYB10)	0.002
245692_at	At5g04150	Basic helix-loop-helix transcription factor (BHLH101)	0.000
261684_at	At1g47400	Expressed protein with unknown function	0.020
248028_at	At5g55620	Expressed protein with unknown function	0.000
251987_at	At3g53280	Cytochrome P450 monooxygenase (CYP71B5)	0.022
264751_at	At1g23020	Fe^3+^-chelate reductase (ATFRO3)	0.008
262218_at	At1g74770	Zinc finger (C3HC4-type RING finger) family protein	0.016
251545_at	At3g58810	Member of the zinc transporter (ZAT) family (ATMTPa2)	0.048
248270_at	At5g53450	ORG1 (OBP3-RESPONSIVE GENE 1), serine/threonine protein kinases	0.000
250828_at	At5g05250	Expressed protein with unknown function	0.000
262412_at	At1g34760	14-3-3 protein GF14 omicron	0.005
259228_at	At3g07720	Expressed protein with unknown function and containing a kelch repeat	0.044
262217_at	At1g74770	Expressed protein with unknown function and containing a Zn binding domain	0.005
251293_at	At3g61930	Expressed protein with unknown function	0.044
257135_at	At3g12900	2-OG-Fe^2+ ^oxygenase family protein	0.039
252502_at	At3g46900	Copper transport (COPT2)	0.012
254550_at	At4g19690	Fe^2+ ^transport protein (IRT1)	0.039

^a ^Listed are the genes that showed a significantly (p < 0.05) increased in transcript abundance in response to Fe deficiency and whose intensity was shown in Figure 3 (Cluster 6, upper part). The list was sorted (top to bottom) to corresponds to the order shown in Figure 3. Gene annotation is based on the TAIR 8.0 release of the *Arabidopsis *genome.

The second subgroup in cluster six responded more slowly to Fe deficiency and showed a significant increase in transcript abundance at 24 h of Fe-deficient growth with only slight increases after 6 h. The increases after 6 h Fe-deficient growth were in most cases not significant (*p *> 0.05). The behavior of this subgroup to Fe deficiency was similar to approximately one-third of the transcripts grouped into cluster four (Figure 3). When all clusters with a significantly increased abundance under Fe deficiency were re-clustered, the group belonging to cluster four transcripts was associated with cluster six (Figure 3). The genes responding more slowly to Fe deficiency and belonging to cluster six are listed in Table 4 and those belonging to cluster four in Table 5.

**Table 4:** List and description of genes transcripts weakly up-regulated by Fe deficiency^a^

Probe ID	ATG	Description	*p*-value
257062_at	At3g18290	Zinc finger protein, C3HC4 type (RING finger)	0.011
264506_at	At1g09560	Germin-like protein (GLP5)	0.027
250952_at	At5g03570	Ni/Fe transporter on the vacuole (ATIREG2)	0.049
247001_at	At5g67330	NRAMP metal ion transporter 4 (NRAMP4)	0.000
253937_at	At4g26890	Mitogen-activated protein kinase kinase kinase 16, MAPKKK16	0.002
263985_at	At2g42750	Similar to DNAJ heat shock protein	0.000
262655_s_at	At1g14190	Similar to glucose-methanol-choline (GMC) oxidoreductase family protein	0.005
259433_at	At1g01570	Similar to fringe-related protein	0.001
265582_at	At2g20030	Identical to putative RING-H2 finger protein ATL2D precursor	0.003
266162_at	At2g28160	Basic helix-loop-helix transcription factor (BHLH029)	0.005
252427_at	At3g47640	Basic helix-loop-helix transcription factor (BHLH047)	0.000
251315_at	At3g61410	Similar to protein kinase family protein, U-box domain-containing protein	0.033
251704_at	At3g56360	Expressed protein with unknown function	0.005
262238_at	At1g48300	Similar to soluble diacylglycerol acyltransferase	0.003
250248_at	At5g13740	ZIF1 (ZINC INDUCED FACILITATOR 1); carbohydrate transmembrane transporter	0.009
245962_at	At5g19700	MATE efflux protein-related	0.041

^a ^Listed are the genes that showed a significantly (p < 0.05) increased in transcript abundance in response to Fe deficiency and whose intensity was shown in Figure 3 (Cluster 6). The list was sorted (top to bottom) to corresponds to the order shown in Figure 3. Gene annotation is based on the TAIR 8.0 release of the *Arabidopsis *genome.

**Table 5:** List and description of genes transcripts weakly up-regulated by Fe deficiency^a^

Probe ID	ATG	Description	*p*-value
258551_at	At3g06890	Similar to oxidoreductase, transition metal ion binding	0.027
245141_at	At2g45400	Homologous to dihydroflavonol 4-reductase and anthocyanidin reductase (BEN1)	0.007
246847_at	At5g26820	Ferroportin-related; similar to iron-responsive transporter-related	0.006
253709_at	At4g29220	Similar to phosphofructokinase family protein	0.005
252916_at	At4g38950	Kinesin motor family protein	0.018
266808_at	At2g29995	Expressed protein with unknown function	0.003
248781_at	At5g47870	Expressed protein with unknown function	0.017
259472_at	At1g18910	Expressed protein with protein binding/zinc ion binding domains	0.040
254298_at	At4g22890	PGRL1A, a transmembrane protein present in thylakoids.	0.004
265539_at	At2g15830	Expressed protein with unknown function	0.028
254013_at	At4g26050	Leucine-rich repeat protein	0.023

^a ^Listed are the genes that showed a significantly (*p *< 0.05) increased in transcript abundance in response to Fe deficiency. The genes in this group clustered weakly into cluster six or overlapped with cluster four. The list was sorted (top to bottom) to corresponds to the order shown in Figure 3. Gene annotation is based on the TAIR 8.0 genome release.

**Figure 5:**
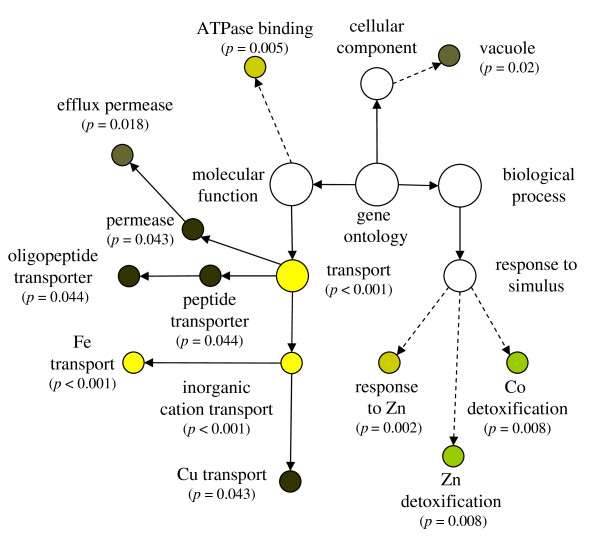
**Over representation of gene ontogeny categories (GO) in genes showing increased abundance under Fe deficiency**. The genes, taken from Table 3–4, were analyzed using BinGO [50] and corrected for false discovery rate using the method of Benjamini and Hochberg [51]. Dotted lines indicate intermediate categories that are not shown.

Summarizing the general behavior of transcripts with increased abundance under Fe deficiency, GO categories related to the transport of Fe, Cu, oligopeptides and amino acids were strongly over-represented (Figure 5). Transcripts annotated to response to Zn and to detoxification of Zn and Co were predominant in this group. Furthermore, cellular components associated with the vacuole were over-represented, indicating the important role of the vacuole in the Fe-deficiency response.

### Analysis of the differential response to Fe deficiency at the transcript level

**Figure 6:**
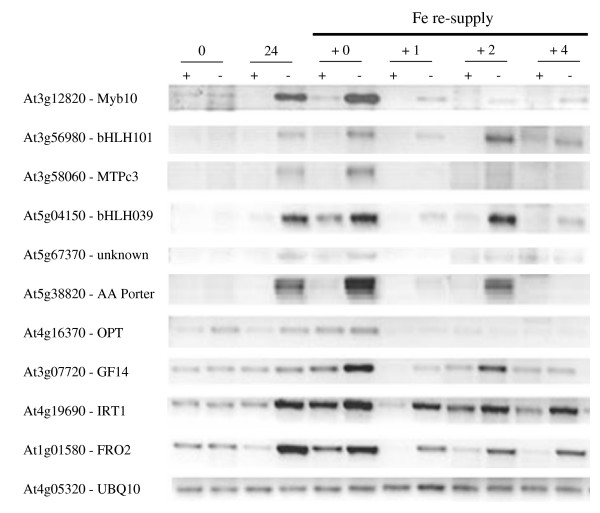
**Semi-quantitative RT-PCR analysis of geneexpression**. Plants were grown for 24 h with or without Fe and transferred to Fe-sufficient medium. Roots were harvested and analyzed by PCR as describe in the Materials and Methods. Nine genes were selected from Table 3 for analysis. RNA abundance in the different treatments was standardized using the ubiquitin 10 gene. The experiment was repeated once with similar results.

The transcripts reported in Table 3 showed the greatest response to Fe deficiency based on fold-induction of any of the transcripts that were identified to be differentially regulated by Fe supply. To confirm the response of a portion of these transcripts to Fe deficiency, the transcript abundance of nine randomly chosen genes from Table 3 was analyzed by semi-quantitative PCR following Fe-deficient growth for 24 h and following Fe re-supply. As a well documented Fe response gene, *FRO2 *was included in the analysis for reference. In agreement with the published data, *FRO2 *and *IRT1 *were strongly up-regulated at 24 h following the removal of Fe from the growth medium (Figure 6). With the re-supply of Fe, transcript abundance for these genes remained elevated over the subsequent 4 h compared to the Fe-sufficient controls. In two cases, At5g67370 and At4g16370, the response to Fe deficiency was generally weak and a clear response was not consistently observed. All other genes tested were strongly up-regulated under conditions of Fe deficiency, thus confirming the results of the microarray experiments. Following the re-supply of Fe, the transcript abundance of these individuals decreased rapidly after 1 h of re-supply, and in most cases no or only weak signals were observed after 4 h of Fe re-supply.

**Figure 7:**
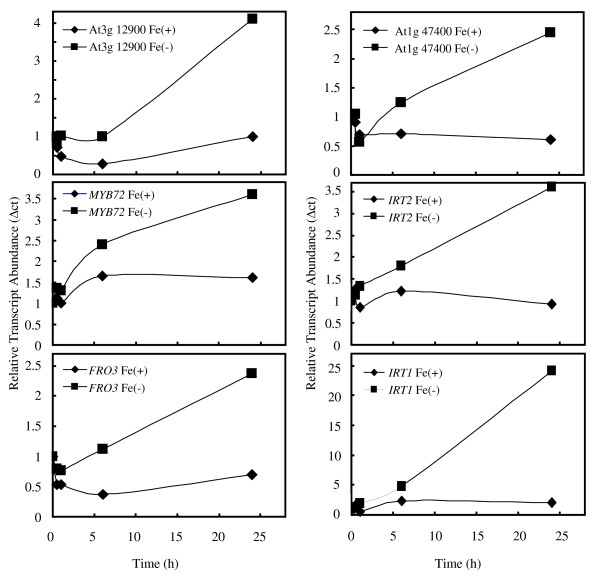
**Real-time RT-PCR analysis of gene expression**. Plants were transferred to either Fe-deficient or Fe-sufficient media and transcript abundance was monitored at times 0, 6 and 24 h following the transfer. Roots were harvested and analyzed by qRT-PCR as described in the Materials and Methods. Six genes were selected from Table 3 for analysis. RNA abundance in the different treatments was standardized using the α-tubulin (At5g19770) gene. The experiment was repeated twice with similar results.

A quantitative validation of the microarray experiments was performed by real-time RT-PCR. The time-course of expression of six genes identified as being strongly induced by Fe deficiency (Table 3) was monitored over the experimental period (Figure 7). The changes in transcript abundance corresponded well with the Gene Chip signals, thus validating the data obtained from the microarray experiments.

## Discussion

### Transcript abundance changes rapidly in response to Fe deficiency

The general response of plants to Fe deficiency has been well documented, particularly with regards to *Arabidopsis *and rice [3]. It is still, however, largely unclear how the Fe status of the plant is perceived and which cellular components might be involved in signal perception. In many of the previous studies of gene expression during the response to Fe deficiency in *Arabidopsis*, plants were cultivated on agar plates and transferred to plates lacking Fe and often supplemented with a ferrous chelator to induce the response to Fe deficiency. In our opinion, the disadvantages to this approach are three fold. First, the agar plate method of induces more mechanical damage to the roots as a result of transfer, second, the inevitable contamination of a variable amount of Fe-containing agar that was carried over during transfer, and last, the harsh side effects of extraction with ferrous chelates. In order to overcome these adverse effects, we have carried out the experiments using a hydroponics system that has been employed previously [28]. This system allows simultaneous transfer of a large number of plants to Fe-free media with little mechanical damage to the root system. Our current analysis of global gene expression during the early phases of the Fe-deficiency response demonstrated that the changes in transcript abundance relevant to the adaptive Fe stress response are occurring at the 6 h time point and time points following (Table 1 and Figure 1). In preliminary analyses that formed the basis of the investigation presented here, we determined the transcript abundance of representative genes that are known to respond to Fe deficiency (e.g. *IRT1*, *FRO2 *and *FRO3*). The result of that study showed that although changes in transcript abundance of Fe responsive genes could be observed at time-point prior to 6 h, the response at and prior to 4 h was variable and the changes in transcript abundance that were observed did not persist (data not shown). Differential expression was observed immediately following transfer and after 0.5 and 1 h of growth in Fe-depleted hydroponic culture media. However, these changes were restricted to individual time-points and the change were not continuous between the early time-points. In fact, a large overlap in significantly changed gene expression was only observed between 6 and 24 h Fe-deficient growth (Figure 1).

The results from our study correspond well with a previous microarray analysis on the *fit *mutant by Colangelo and Guerinot [16]. Of the 72 genes that were identified by the previous study as being responsive to Fe deficiency with increased transcript abundance, 22 were also found in the present study. These 22 genes were distributed between early and late responding transcripts.

### Rapid transcriptional changes are associated with Fe uptake and distribution

Considering the Fe deficiency-dependent and time-dependent changes in transcript abundance, we have identified 16 genes whose abundance was decreased and 51 whose abundance was increased over the 24 h period. The decreased abundance in transcripts encoding two nodulin-like proteins (At3g25190 and At1g21140) was of particular interest, because these proteins contain a domain belonging to the CCC1-like family. The family of proteins with this domain includes a yeast vacuolar transmembrane protein (CCC1) that has been shown to transport Fe and Mn from the cytosol into vacuole [30] and VIT1, a CCC1 homolog in *Arabidopsis *that has been shown to be an influx Fe transporter on the vacuolar membrane [31]. If the function of the two nodulin-like proteins were similar to VIT1, then the decreased abundance of these transcripts would be associated with a decreased capacity of the cell to store Fe in the vacuole. The decreased abundance in the three transcripts encoding ferritins was also easily understood in the context of Fe mobilization in response to deficiency.

Somewhat puzzling was the presence of three transcripts encoding chloroplast proteins in the group of most significantly down-regulated transcripts (Table 2; At3g26650, At2g30570 and At2g05070). At3g26650 encodes one of the two subunits of the chloroplast glyceraldehyde-3-phosphate dehydrogenase (GAPDH). This gene has been shown to be coordinately expressed with the phosphoribulokinase gene (PRK) and CP12-2 genes; however, in our study the three CP12-2 and the PRK genes showed no significant changes in response to Fe deficiency (data not shown). At2g30570 encodes a protein similar to subunit W of the photosystem II reaction center, and At2g05070 encodes Lhcb2.2, a member of the family of light-harvesting chlorophyll a/b-binding (LHC). How these transcripts might be involved in the root response to Fe deficiency is not known.

In contrast to the genes that displayed decreasing transcript abundance in response to Fe deficiency, gene which showed an increased in abundance formed a larger more heterogeneous group. They were subdivided further based on cluster analysis. One of these groups responded more rapidly the Fe deficiency (Table 3) than the other (Tables 4 and 5). As stated above, genes encoding Fe transporters, transcription factors and Fe-containing proteins were prominent among these up-regulated transcripts. With regard to metal transporters, the most rapidly responding transcripts were those encoding the Fe uptake transporters (IRT1 and IRT2 [9,32]), the Fe^3+^-chelate reductase FRO3 [33], and the oligopeptide-Fe transporter OPT3 [34]. The transcript abundance of NRAMP4, a vacuolar Fe efflux carrier [35], and of ferroportin, a putative Fe efflux carrier of unknown location [36], was increased at the 24 h time point but not before. Thus, based on the results of the present study, we suggest that the response of *Arabidopsis *to Fe deficiency was to increase and maintain the capacity for Fe uptake in the cytoplasm.

Recently, an investigation of the transcriptional response of *Arabidopsis *roots to abiotic stress, including the response to Fe deficiency, has been performed by Dinneny et al. using a cell sorting approach to increase the resolution of the analysis [37]. Upon reprocessing the microarray data published in that study using the methods and criteria concurrent with our own investigation, it was revealed that the number of transcripts which show a significant change was greater than 3100, in contrast to less than 100 transcripts in the present study. While a considerable overlap was also found between the two studies, the individuals identified by Dinneny et al. [37] generally appeared after a longer exposure to Fe deficiency in the present study. In contrast, the differences in the respective studies can most likely be attributed to the different method of culturing the plants used in the experiments. However, possibly as a result of the high resolution of their systems, the majority of the transcripts identified in the Dinneny et al. [37] study appears to have no clear connection to the known responses to Fe deficiency and may represent, at least partly, biological signals unrelated to the Fe-deficiency response.

### Fe deficiency induces rapid changes in Zn homeostasis

In addition to regulation of Fe transporter abundance under Fe deficiency, transcripts corresponding to Zn transporters were regulated under Fe deficiency. In this case, transcripts for the Zn plasma membrane efflux carrier, MTPc3 [38], and the vacuolar influx carrier, MTPa2 [39], were strongly and rapidly increased, while transcripts for the Zn plasma membrane influx carrier, ZIP3, were decreased. At the 24 h time-point, transcripts for the Zn vacuolar influx carrier, ZIF1 [40], were also increased. The effect of Fe deficiency was not restricted to Fe and Zn. A Cu transporter [41] was rapidly increased and the Fe-regulated Ni transport was slowly increased [42]. ZIP3 (At2g32270) has been shown to be a Zn transporter that catalyzed Zn uptake when heterologically expressed in yeast [43]. ZIP3 responded to Zn deficiency [43], but a role for ZIP3 in Fe transport has not been shown.

## Conclusion

**Figure 8:**
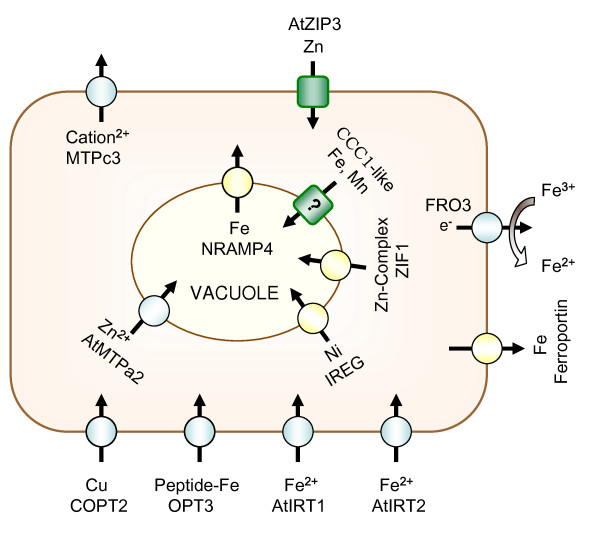
**Summary of the immediate changes in ion transport in response to Fe deficiency**. Squares were used to depict transporters with decreased and circles transporters with increased abundances. Yellow symbols represent changes that were observed only after 24 h of Fe deficiency, whereas the grey symbols were observed at both 6 and 24 h. The genes encoding the proteins shown are: *COPT2 *(At3g46900, [41]), *OPT3 *(At4g16370, [34]), *IRT1 *(At4g19690, [9]), *IRT2 *(At4g19680, [32]), Ferroportin (At5g26820, [36]), *FRO3 *(At1g23020, [33]), *ZIP3 *(At2g32270, [43]), *MTPc3 *(At3g58060, [38]), *MTPa2 *(At3g58810, [39]), *IREG2 *(At5g03570, [42]), *ZIF1 *(At5g13740, [40]), *CCC1*-like (At3g25190 [30] and *NRAMP4 *(At5g67330, [35]). The subcellular localization has been experimentally demonstrated for COPT2, IRT1, MTPa2, IREG2 and NRAMP4. Localization of the other proteins is hypothetical and was assumed according to their predicted function.

A summary of the effects of Fe deficiency on ion transport in the cell is presented in Figure 8. Assuming that the regulation of transcript abundance is also reflected in altered ion fluxes in the cell, the response of the cell is to increase cytosolic Fe through increased influx into the cytosol and decreased efflux into the vacuole. On the other hand, rapid regulation of the gene transcripts MTPc3 and MTPa2 indicates changes associated with divalent cation homeostasis and have the effect of decreasing divalent cation influx into and increasing efflux out of the cytosol. The response of the cell to an increase cytosolic Fe concentration under conditions of Fe deficiency could be easily understood, whereas the opposite reaction to a reduction in the cytosolic Zn concentration is more difficult to explain. Perhaps under Fe deficiency the capacity for Zn uptake exceeded the nutritional requirement of the cells, and the cytosolic uptake as well as the efflux capacity was adjusted to establish a new Zn homeostasis. The specificity of the plant metal transporters may be the molecular cause for the deregulation of metal ion homeostasis during Fe deficiency. Recently, it was reported that a correlation between Fe deficiency and increased shoot concentrations of Zn, Mn and Co [44]. Fe-deficient *Arabidopsis *roots accumulated high concentrations of Co, Mn and Zn compared to nutrient sufficient controls [9,10,45]. Thus, a side effect of increasing the Fe uptake capacity is a concomitant uptake other metals via the IRT1 transporter, when the availability of Fe in the media is low.

The re-supply of Fe to Fe-deficient plants did not in all cases lead to a rapid down-regulation of genes which are highly induced by Fe-deficiency. In some cases, e.g. for bHLH101 and bHlH39 re-induction was observed 2 h after Fe resupply after an initial repression by Fe after 1 h. Such an effect has been previously described for IRT1 and FRO2 and attributed to in the regulation of Fe-responsive genes by both local and systemic signals [46]. Our data demonstrate that this regulation is not restricted to IRT1 and FRO2, but also include genes with regulatory function.

## Methods

### Plant Material and Growth Conditions

*Arabidopsis thaliana *L. cv. Landsberg erecta was grown hydroponically in a growth chamber at a constant relative humidity of 75%, under a 10/14 h light/dark cycle (Osram E40/ES Plantastar, 300 μmol cm^-2 ^s^-1^) at 21°C (day) or 18°C (night). Two to four seeds were sown in 1.5 ml Eppendorf^® ^tubes that were filled with 1 ml of 0.25% agarose. The tip of the tube was cut off with a hot scalpel and the tubes were placed in black-plastic containers in contact with the nutrient solution that was constantly aerated. Each container contained approximately 45 Eppendorf^® ^tubes. At 20 days following sowing, the agarose plug was removed using a gentle stream of water. The nutrient solution was replaced weekly and at 24 h prior to beginning the experiment. The nutrient solution was composed of KNO_3 _(3 mM), MgSO_4 _(0.5 mM), CaCl_2 _(1.5 mM), K_2_SO_4 _(1.5 mM), NaH_2_PO_4 _(1.5 mM), H_3_BO_3 _(25 μM), MnSO_4 _(1 μM), ZnSO_4 _(0.5 μM), (NH_4_)_6_Mo_7_O_24 _(0.05 μM) CuSO_4 _(0.3 μM), Fe-EDTA (40 μM) with pH adjusted to 6.0 with KOH [47].

At 30 days after sowing, the plants were washed briefly in a 0.1 mM EDTA solution and transferred into either Fe-free or Fe-replete (controls) nutrient solutions. Following transfer, the roots were harvested after 0, 0.5, 1, 6 and 24 h into RNALater^® ^(Applied Biosystems). The experiment was initiated 1 h into the light cycle. For each time-point analyzed, approximately 20 plants were harvested. The total processing time was approximately 15 s. To confirm that the plants were responding to Fe deficiency, the Fe^3+^-chelate reductase activity was determined after 24 h Fe deficiency using the method of Moog et al [48]. Only groups of Fe-deficient plants that showed a greater than 80% increase in the Fe^3+^-chelate reductase at 24 h were used for RNA isolation. The data reported are averages taken from three independent experiments.

In the re-feeding experiments shown in Figure 6, 30-day-old plants were washed in 0.1 mM EDTA as described above and transferred for 24 h to Fe-deficient media. Subsequently, samples were taken before transfer (0 h control), following 24 h growth in Fe-deficient media (24 h -Fe) and following resupply of Fe at 0, 1, 2 and 4 h. Control plants were treated similarly but were grown in the presence of Fe.

### RNA Preparation and DNA-Microarray Hybridization

Total RNA was extracted from roots (RNeasy^® ^Midi Kit, Qiagen). The microarray analysis was performed using the Affymetrix ATH1 *Arabidopsis *GeneChip. The quality control of RNA, preparation of cRNA and hybridization of oligonucleotide chips was performed by the Affymetrix Gene Expression Service Lab. at the IPMB, Academia Sinica .

### Preprocessing and Statistical Analysis

Preprocessing and statistical analysis of data was performed using the Bioconductor packages AffylmGUI and Limma ([49]). Arrays were normalized using the Robust Probe-level Linear Model (PLM) provided in the AffylmGUI package using the default parameters. Over- and under-represented gene ontogeny categories (GO) were determined using the program BiNGO 2.3 with Cytoscape 2.6 ([50]) and the website Amigo . The correction for false discovery rates was determined with Bingo software using the method of Benjamini and Hochberg [51]. Cluster analysis was performed using the k-means methods with the algorithm of Hartigan and Wong [29]. The cluster stability was determined using the "benhur" function found in the clusterStab program of Bioconductor .

All microarray data from this study have been deposited in NCBI Gene Expression Omnibus (accession number GSE15189).

### Semi-Quanitative RT-PCR

cDNA was synthesized from one μg total RNA using an oligo-dT primer. The abundance of cDNA was standardized to the abundance of ubiquitin 10. The amount of cDNA was visualized by ethidium bromide staining following agrarose gel electrophoretic separation. The PCR primers for the genes tested were: AA-Transporter (At5g38820) fwd: ctacgcagctcatctcatgc, rev: cgattgcattggagaaaacc, bHLH039 (At3g56980) fwd: gctttggtgtttctgtttcg, rev: tcacttcgttgtcaccaagc, bHLH101 (At5g04150) fwd: ccgccgtagaaaacttaacg, rev: gacgtagcaatctggacagc, cation flux (At3g58060) fwd: ttcgtcaaacgatcatgagg, rev: tcttgtgcagctctctcagc, CYP71B5 (At3g53280) fwd: agaattggcgagagatgagg, rev: tggaaaaggctttcatcacc, FRO2 (At1g01580) fwd: cctaaatcagctgccgcacatgacg, rev: gttcacaaacattatgctcgtcgggc, IRT1 (At4g19690) fwd: ccccatggtcatggtcatgg, rev: attccaccgcacccgagaag, kelch-repeat (At3g07720) fwd: taatttgcagcagagcaacg, rev: ttgagctcctggtgagtcg, Myb10 (At3g12820) fwd: caaaattggcgatctcttcc, rev: tttccccaaactcctcttcc, Myb72 (At1g56160) fwd: gaaaggaagagcaccatgc, rev: tttcccacatctcaacaatcc, OPT3 (At4g16370) fwd: ggaatgctctttgcttttgc, rev: gggctattagggtggtctcc, ubiqutin (At4g05320) fwd: ggaaaacaattggaggatgg, rev: ttagaaaccaccacgaagacg, unknown (At1g47400) fwd: gtcttttgtcgcaaacttgg, rev: taggaaacaatcacgcagca, unknown (At5g55620) fwd: ccaaatgttcggctactcg, rev: ttgatatgcatgtggagagc, unknown (At5g67370), fwd: ggtttgacccacatttgtcc, rev: tgagtttctcgggtcaaagc, Zn binding (At1g74770) fwd: atgtttgcagggaaaagtgc, rev: gtaggagccacaggttgtgc

### Real-time RT PCR

Total RNA was isolated from roots of 20 plants with RNeasy Plant Mini Kit (Qiagen) according to the manufacturer's instructions. Nucleic acid quantity was evaluated by using a NanoDrop ND-1000 UV-Vis Spectrophotometer (NanoDrop Technologies, Wilmington, USA). One μg of total DNase-treated RNA (Turbo DNase, Ambion) was reverse-transcribed using Superscript III Reverse Transcriptase (Invitrogen) with oligo dT primers in a total volume of 20 μL. Real-time quantitative PCR was performed using double-stranded DNA binding dye Syber Green PCR Master mix (Applied Biosystems) in an ABI GeneAmp 7000 Sequence Detection System. Each reaction was run in a triplicate and the melting curves were constructed using Dissociation Curves Software (Applied Biosystems), to ensure that only a single product is amplified. Validation experiments were performed to test the efficiency of the target amplification and the efficiency of the reference amplification. Duplicate C_T _values were analysed with Microsoft Excel using the comparative C_T_(ΔC_T_) method as described by the manufacturer (Applied Biosystems). The amount of target (2^-ΔΔC^_T_) was obtained by normalizing to an endogenous reference (α-tubulin, At5g19770) and relative to a calibrator (control tissue). The PCR primers for the genes tested were: FRO3 (At1G23020) fwd: ccatcactcctcaatcacttcca, rev: tccagccttgcttgccata, IRT1 (At4g19690) fwd: caccattcggaatagcgttagg, rev: ccagcggagcatgcattta, IRT2 (At4g19680) fwd: cgtatctccggcgatctca, rev: tgatgcacgggttatcaaagc, MYB72 (At1G56165) fwd: cgagaggtaaccaaatcgcaat, rev: tcttcaaatccgcatgggata, At3G12900 fwd: ccagcctctctctgagcgaat, rev: ctgggtagcctcacacgtaagg, At1G47400 fwd: ggcatatagtgagaatggtggtgat, rev: tcacgcagcaggagcataat

## Authors' contributions

TJB led the experimental design of the present study, carried out the data analysis and drafted the manuscript. TJWY contributed to the design of the study, prepared RNA samples, conducted experiments to validate the microarray data, and was involved in editing of the manuscript. WS participated in the data analysis, strategic planning of the work, and writing of the manuscript. All authors read and approved the final manuscript.
